# pyseer: a comprehensive tool for microbial pangenome-wide association studies

**DOI:** 10.1093/bioinformatics/bty539

**Published:** 2018-07-02

**Authors:** John A Lees, Marco Galardini, Stephen D Bentley, Jeffrey N Weiser, Jukka Corander

**Affiliations:** 1 Department of Microbiology, New York University School of Medicine, New York, NY, USA; 2 European Bioinformatics Institute (EMBL-EBI), Hinxton, UK; 3 Department of Parasites and Microbes, Wellcome Sanger Institute, Hinxton, UK; 4 Department of Biostatistics, University of Oslo, Oslo, Norway; 5 Helsinki Institute of Information Technology (HIIT), Department of Mathematics and Statistics, University of Helsinki, Helsinki, Finland

## Abstract

**Summary:**

Genome-wide association studies (GWAS) in microbes have different challenges to GWAS in eukaryotes. These have been addressed by a number of different methods. *pyseer* brings these techniques together in one package tailored to microbial GWAS, allows greater flexibility of the input data used, and adds new methods to interpret the association results.

**Availability and implementation:**

*pyseer* is written in python and is freely available at https://github.com/mgalardini/pyseer, or can be installed through *pip*. Documentation and a tutorial are available at http://pyseer.readthedocs.io.

**Supplementary information:**

Supplementary data are available at *Bioinformatics* online.

## 1 Introduction

Finding genetic variation associated with bacterial phenotypes such as antibiotic resistance, virulence and host specificity has great potential for a better understanding of the evolution of these traits, and may be able to inform new clinical interventions. Genome-wide association studies (GWAS) address this in a hypothesis-free manner. The recent availability of thousands of whole-genome sequences from bacterial populations has made this approach possible, though issues of strong clonal population structure and a variable pangenome (sequence variation of both core regions present in every isolate, and in variably present accessory regions) must be accounted for in any successful analysis (Power *et al.*, 2017).

One method that addresses these issues in a scalable manner is *SEER* (Lees *et al.*, 2016), which uses non-redundant variable length k-mers between 9-100 bases as a generalized variant to represent variation across the pangenome. Linear models with a control for population structure are then used to perform the association. Other methods include: *bugwas*, which uses a linear mixed model (LMM) and also checks for lineage effects (Earle *et al.*, 2016); *scoary*, which tests clusters of orthologous genes (COGs) with and without accounting for population structure (Brynildsrud *et al.*, 2016); and phylogenetic regression, which uses a known phylogeny to adjust for the covariance between samples (Garland and Ives, 2000).

Each of these methods has its own set of advantages and limitations, so recent GWAS analyses have used a combination of these techniques along with methods designed for human genetics, often in a somewhat ad-hoc manner that requires tedious file format conversion and familiarity with a wide range of methods and software packages (Lees *et al.*, 2017).

As the number of large bacterial population datasets increases, we recognize the need to make these methods more accessible to the field. We have therefore re-implemented *SEER* in an easy-to-use and install *python* package, *pyseer*. *pyseer* also includes many additional features covering new association models, input sources and output processing. This brings together all the methods mentioned above into a single piece of software, and further enables new combinations of analysis. Our method is equally applicable to viral GWAS, and indeed any species with structured populations and/or genomes with significant variation in gene content.

## 2 Materials and methods

The foundation of *pyseer* is a direct *python* re-implementation of *SEER* (originally written in C++). K-mers of variable length counted from draft assemblies are used as the input, and their association with a phenotype of interest is assessed by fitting a generalized linear model to each k-mer. To control for population structure, multi-dimensional scaling of a pairwise distance matrix is performed and these components are included as fixed effects in each regression. In the case of a binary phenotype with a highly penetrant variant (those with high effect sizes), Firth regression is performed to maintain power. This adds a penalty to the logistic regression likelihood when there is a large error on the slope, which alleviates underestimation of p-values in cases with nearly separable data (Heinze and Schemper, 2002). Significant k-mers, after adjusting for multiple testing, can then be mapped to a reference annotation to find regions of the genome associated with the phenotype.

After re-implementation, results were the same as *SEER* (Supplementary Fig. S1) and *pyseer* is of comparable speed (Supplementary Table S1). We then expanded the features in *pyseer* to bring together the different methods mentioned above.

### 2.1 Input sources

In addition to k-mers, the original focus of *SEER*, it can be convenient to test for association of SNPs and INDELs called against a reference genome. These can be used to test for association of close or adjacent SNPs, which would be split into many low frequency k-mers. The consequence of these variants can be directly predicted (e.g. synonymous, frameshifting) which would require more downstream processing for k-mers. Presence or absence of COGs and aligned intergenic regions can also be useful, as shown by *scoary* (Brynildsrud *et al.*, 2016).

*pyseer* can natively read all these types of variant from VCF or Rtab files, which also allows for any user defined input type (for example copy number variants). We also enable grouping of variants by genomic region to perform burden testing, now allowing users to perform analysis of rare variation while still accounting for population structure.

An important technical difficulty of using *SEER*, and most common linear mixed model implementations, is making sure that the membership and order of samples between the variant calls, phenotype file and population structure all match up correctly. Many methods do not check for this, and can run an incorrect analysis without producing a warning. In *pyseer* this label matching is done automatically, and the intersection of samples used is reported to the user.

### 2.2 Association models

*pyseer* implements the same fixed effect generalized linear regression model as *SEER*, including Firth regression. In *pyseer* we have added two new multidimensional scaling algorithms and a more streamlined interface with *mash* (Ondov *et al.*, 2016) to compute population structure. Alternatively, when a high quality phylogeny is available, *pyseer* can use this to adjust for population structure in a manner analogous to phylogenetic regression.

As a major alternative we have included an LMM, which uses random effects to control for population structure, and has been shown to work across a range of scenarios. We have used the FaST-LMM implementation, which allows association in linear time (Lippert *et al.*, 2011), using a kinship matrix estimated from a subset of variants or from a phylogeny. This now allows association of all k-mers under the mixed model in a few hours, which was previously computationally and bioinformatically challenging.

We have also included a method to estimate possible lineage effects, based on the procedure used in *bugwas*. Those variants which are associated with both the phenotype and with a lineage (which can be determined by *pyseer* or defined by the user) associated with the phenotype can be prioritized for further analysis outside of GWAS.

### 2.3 Output processing

It has been suggested that the number of unique variant patterns is a sensible way to set the multiple testing threshold, however these can be difficult to count due to the size of k-mer data. *pyseer* uses hashing to efficiently calculate this. We have also added tools to make interpretation of significant k-mers more streamlined. The user can map their results against any number of reference genomes for interactive display in *phandango* (Hadfield *et al.*, 2018) (Supplementary Fig. S2). We have added the ability to summarize k-mer results at the gene level by iteratively mapping to reference and draft annotations, which can be used to show complementary information about effect size, coverage and minor allele frequency (Supplementary Fig. S3).

## 3 Discussion

Starting with a re-implementation of *SEER*, we have added the models and input types used by other microbial and human GWAS approaches into a single package. Analyses which were previously challenging to perform, such as association of all variable length k-mers with a LMM, can be performed in an efficient and user-friendly manner. We have also enabled new types of analysis, such as population structure corrected burden testing, and gene level summaries of k-mers. Our package includes comprehensive documentation and a tutorial which shows how to perform GWAS using the new input sources and both association models, as well as how to interpret significant k-mers. We have implemented unit tests in our code to ensure consistency of output as features are added.

With *pyseer* we have therefore reconciled many of the existing methods for regression-based microbial GWAS into a single package. Our focus on documentation and ease-of-use of *pyseer* will make GWAS more accessible to the microbial genomics community.

## Supplementary Material

Supplementary Material
