# Supplementary material for: pyseer: a comprehensive tool for microbial pangenome-wide association studies
Source: Bioinformatics. 2018 Jul 2;34(24):4310–2. doi: 10.1093/bioinformatics/bty539 (PMC6289128; doi:10.1093/bioinformatics/bty539)
Supplement: Supplementary Material [file bty539_supplementary_material.pdf]

Supplementary material

Supplementary table 1: Resource comparison between *pyseer* and *SEER* using the tutorial dataset (15.1M k-mers, no filtering).

Association model	Cores	*pyseer*		*SEER*	
		CPU (hrs)	Memory (Mb)	CPU (hrs)	Memory (Mb)
Fixed effects (binary)	1	65.9	90	58.3	490
	4	28.4	450	25.2	500
Fixed effects (continuous)	1	15.3	70	3.6	470
Mixed effects	1	8.1	150	NA	NA
	4	5.8	1600	NA	NA

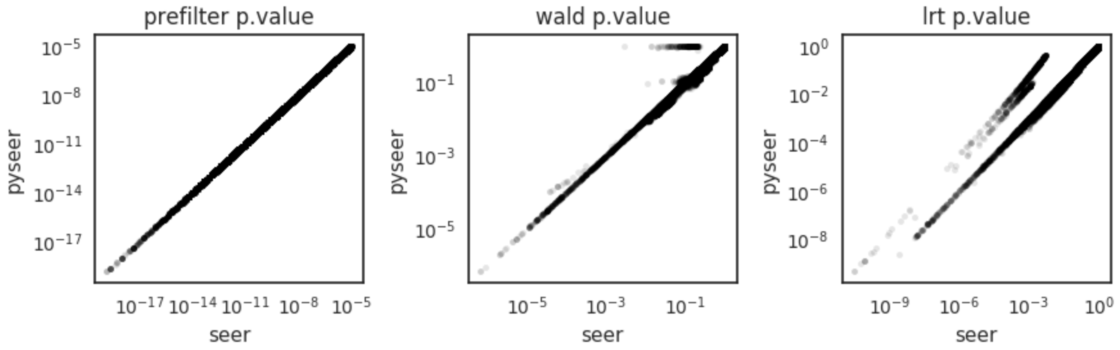

Supplementary figure 1: p-p plot comparing p-values of association between SEER (x-axis) and pyseer (y-axis) on a test dataset. Filter p-values (left) were identical. Wald test p-values (centre) were mostly the same, but we removed this test from pyseer due to its lower power compared to the likelihood ratio test. The likelihood-ratio test p-values (right) revealed an error in the likelihood of Firth regression in SEER, which has been fixed in pyseer. Otherwise, p-values were the same within precision of the normal distribution cdf used to calculate them.

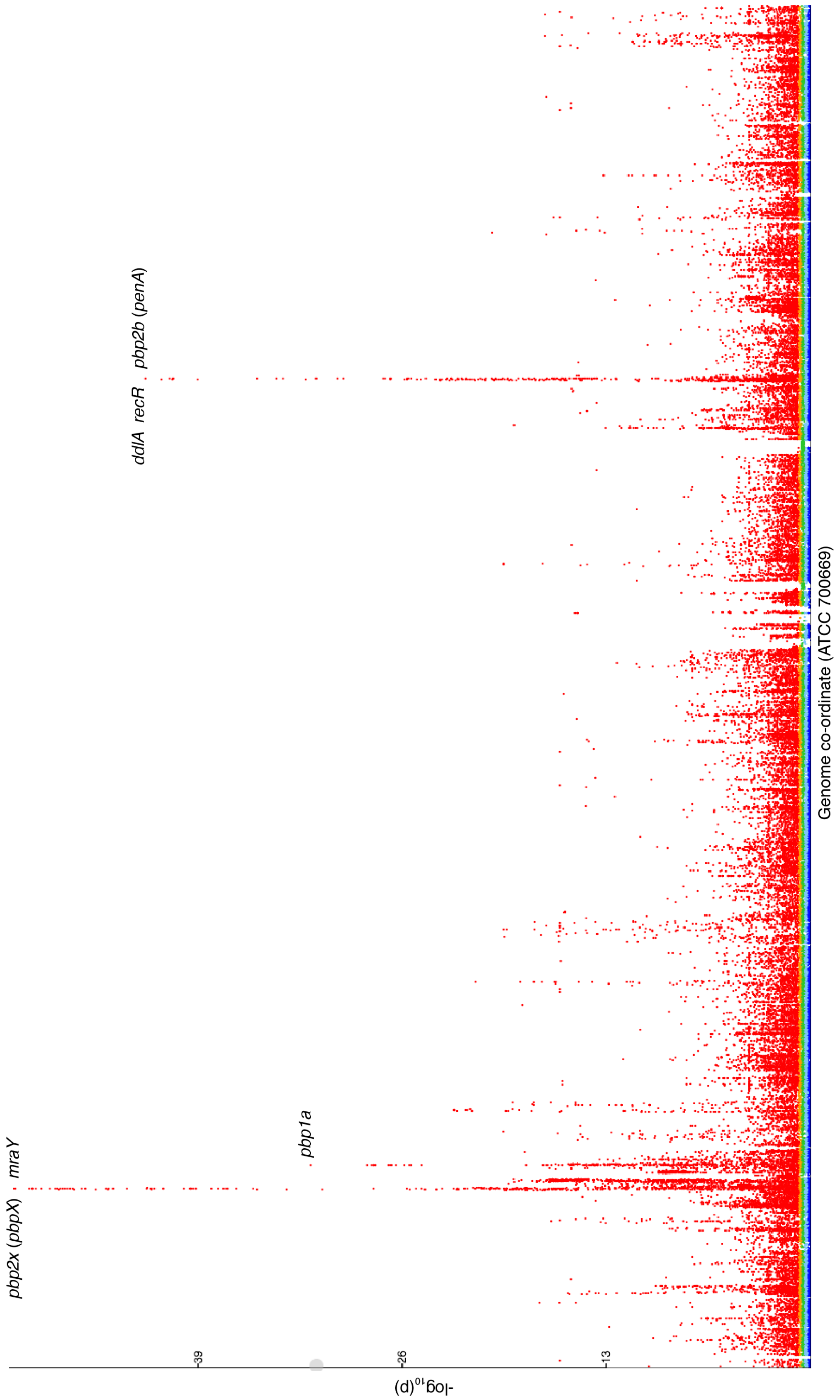

Supplementary figure 2: An example Manhattan plot from a SNP-based GWAS of penicillin resistance in the tutorial dataset using *S. pneumoniae* (http://pyseer.readthedocs.io/en/version2/tutorial.html). We produced the plot using the fixed effects model in pyseer, and visualised the results using phandango.

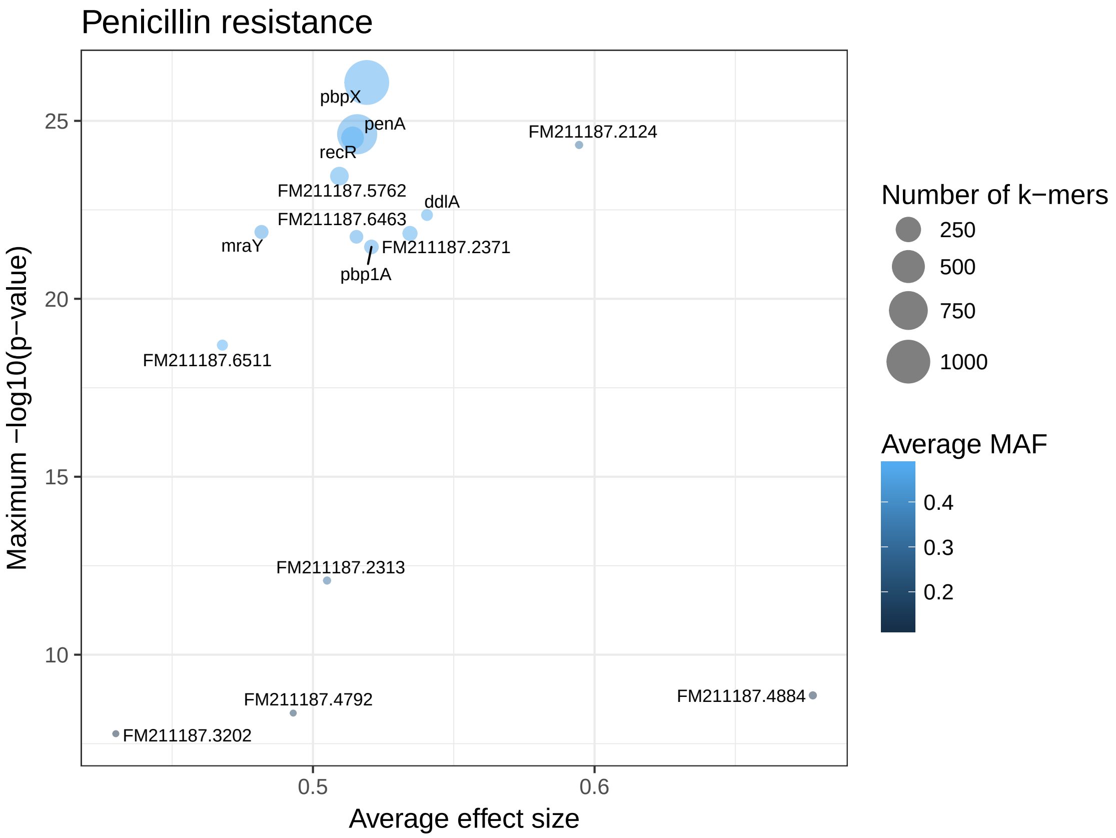

Supplementary figure 3: An example gene-based summary plot from the same data as the Manhattan plot fig. 2. The maximum p-value of association for each gene is shown on the y-axis as in the Manhattan plot and the average effect size on the x-axis. The size of each gene corresponds to the number of significant k-mers overlapping it, and the colour to the average minor allele frequency (MAF) of the overlapping significant k-mers. The plot shows a difference between more common results (for example *penA* and *penX*, which are causal) and rarer results (for example FM211187.4884, which is a false positive). *recR* and *ddlA* are in linkage disequilibrium with the top hits due to their proximity in the genome, which is easier to assess using the Manhattan plot.
